# Traumatic Dental Injuries Among 12-15-Year-Old-School Children in Panchkula

**DOI:** 10.5812/atr.18127

**Published:** 2014-03-30

**Authors:** Amandeep Chopra, Manav Lakhanpal, NC Rao, Nidhi Gupta, Shelja Vashisth

**Affiliations:** 1 Department of Public Health Dentistry, Swami Devi Dyal Hospital and Dental College, Panchkula, Haryana, India

**Keywords:** Dental Occlusion, Traumatic, Prevalence, Risk Factors

## Abstract

**Background:**

Traumatic dental injury (TDI) in children and adolescents has become one of the most serious dental public health problems. Despite such a high prevalence of dental trauma, very less attention has been paid to TDI, its etiology, and prevention.

**Objectives:**

To determine the prevalence of anterior tooth traumatic dental injuries in 12-15-year-old school children of Panchkula district, India, and to find any correlation with the cause, gender, extent of overbite as well as over-jet, and previous treatment.

**Patients and Methods:**

A multistage sample of 12-15-year-old school children (n = 810) in Panchkula district, Haryana, was selected. The children were screened using WHO criteria for oral examination and a trained dental surgeon examined the children. Those with clinical TDI were examined further for the type of traumatic injuries using Elis classification modified by Holland. Overjet and overbite were recorded. After examination, questions regarding the cause of trauma and its treatment were asked. Data were subjected to statistical analysis using the Chi square and Mantel-Haenszel tests by SPSS version 20.0.

**Results:**

The results showed that out of 810 children, 86 (10.2 %) had TDI. Males had higher prevalence of trauma than females (P < 0.05). The common cause of trauma was fall (51.11%) followed by sports injuries (41.86%). Enamel-dentin fracture without pulpal involvement was the most common type of trauma and the most frequent involved teeth were maxillary central incisors. A significant association was observed between overjet and overbite and trauma. Only 3.5% of the children affected with trauma had received treatment.

**Conclusions:**

The prevalence of traumatic injuries to permanent incisors in 12-15-year-old Panchkula school children was relatively high. TDI was associated with gender, overjet, and lip competence. There was a great unmet treatment need.

## 1. Background

Traumatic dental injury (TDI) in children and adolescents has become one of the most serious dental problems of the public health (1). They occur commonly and affect approximately 20-30% of the permanent dentition worldwide that often lead to functional, aesthetic and psychological disturbances as well as significant child, parents, and dentists concerns (2). With regard to TDI, a wide range of prevalence (4.1- 58.6%) from different countries has been reported (3-8). 

Despite such a high prevalence of dental trauma, very little attention has been paid to TDI as well as its etiology and prevention. Risk evaluation for dental trauma is not performed during the routine dental examination even for those who participate in various sports. Since the majority of these injuries are preventable, there is a rising consensus on TDI. Establishing educational programs for children, parents, and teachers concerning the importance of early treatment for dental trauma, ways of preventing these traumas, and procedures for appropriate emergency management are of significant importance. These educational programs for the public in a country should preferably be preceded by an investigation of background information on the occurrence of orodental injuries in the community. Anterior tooth trauma was studied previously and studies reported them as the most susceptible tooth to TDI (9). 

## 2. Objectives

We aimed to evaluate prevalence of TDI involving permanent anterior teeth in 12-15-year-old school children of Panchkula district and to find any correlation with the cause, gender, lip coverage, the extent of overjet, and any previous treatment.

## 3. Patients and Methods

This cross-sectional study was conducted over a period of six months from April 2013 to September 2013 among 12-15-year-old schoolchildren of Panchkula District, Haryana, India. Ethical clearance was obtained from the institutional ethic committee and oral informed consent was obtained from the participants. A pilot study was conducted one month prior to the original study with a sample of 80 individuals who were not part of the main sample. Prevalence of TDI was found to be 8.8%. Based on the results of the pilot study, sample size was calculated 810.

A multistage sampling technique was adopted to select the children. The primary sampling unit consists of four blocks of Panchkula (i.e. Pinjore, Raipur Rani, Barwala, and Morni). Within each block, the schools were randomly selected proportional to number of private and governmental schools and the total number of children going to school. Before examining the children, the consent was obtained from the concerned authorities of education department and principal of respective schools of the Panchkula District. On the day of examination, all the children with the age of 12-15 years who were available in the selected schools were examined until the desired sample size was achieved.

### 3.1. Inclusion Criteria

The children who had entered 12th through 15th year on their last birthday and had erupted permanent anterior teeth were included in the study.

### 3.2. Exclusion Criteria

The children with the following conditions were excluded from the study: Children who were undergoing or had finished orthodontic treatment, children in whom the permanent anterior teeth had not yet erupted, the permanent anterior teeth were lost due to caries or any cause other than trauma, partial/complete anodontia involving permanent anterior teeth, physically-challenged children, cleft lip or palate, or those did not tend to participate.

The examiner was trained prior to the study commencement. A pre-survey calibration was performed on a group of 30 schoolchildren with the age of 12-15 years who were selected from the School Oral Health Program conducted by Dental Institution. The obtained results were subjected to kappa statistics. The calibration exercise and the kappa value (0.95) showed good agreement for these observations and measurements in terms of intra examiner variability, which validated the examination procedure. Dental examination was conducted using individually wrapped and sterilized sets of plain mouth mirrors, community periodontal index probes, and gauze pads. The children were examined at their schools under natural light. Because radiographic examination was not performed, root fractures were not recorded. Pulp vitality tests were not performed. The dental examination for TDI included only anterior permanent teeth. Injuries were classified according to the epidemiological classification given by Ellis and modified by Holland et al. (10). Measurement of maxillary overjet was made with the teeth in centric occlusion; the distance from the labial-incisal edge of the most prominent maxillary incisor to the labial surface of the corresponding mandibular incisor was measured using the Community Periodontal Index (CPI) probe, as described in the 1997 World Health organization (WHO) basic oral health survey guidelines.

Lip coverage was recorded on visual inspection as adequate if lips covered the maxillary incisors in rest position and as inadequate if two-thirds of the crown height was exposed and visible. Demographic information and questions regarding cause of trauma and treatment was recorded as part of the clinical examination. The data were analyzed by SPSS version 20.0 (SPSS Inc., Chicago, IL, USA). Data analysis included descriptive and analytic statistics. The Chi square test was used to compare qualitative data. The strength of association of the variable (lip coverage, gender, and incisal overjet) with the outcome was assessed using the Mantel–Haenszel Common Odds Ratio. The level of statistical significance was set at P < 0.05.

## 4. Results

Out of the 810 students who were examined and responded the questionnaire, 415 were male and 395 were female. The prevalence of TDI was 10.2% (86) with 60 male and 26 female-affected students. Overall, 94.2% of the injured children had only one tooth damaged. 

**Figure 1.**
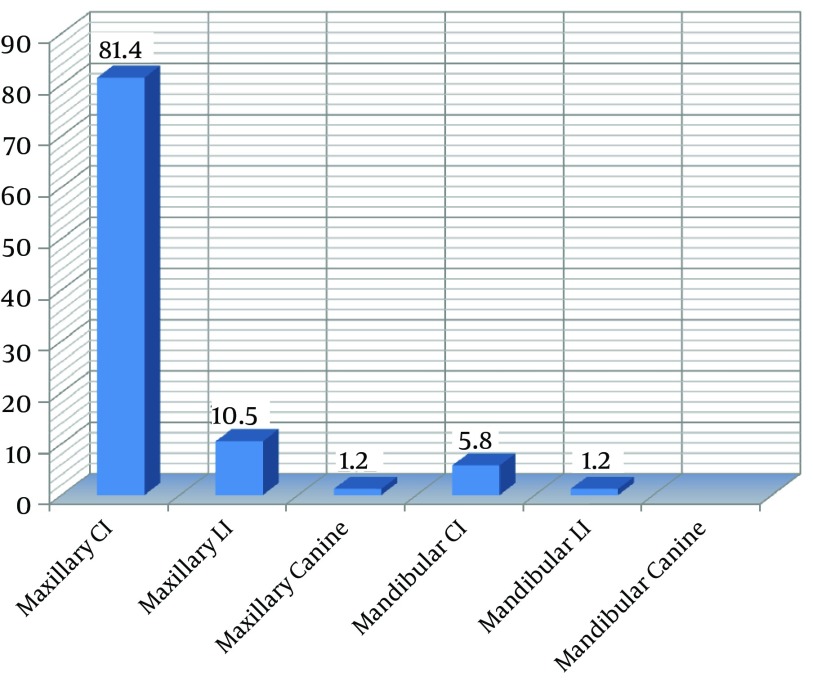
Traumatic Dental Injuries Classified According to Type of Teeth Injured

The maxillary central incisors were the most frequently affected teeth (81.4%), followed by the maxillary lateral incisors (10.5%), the mandibular central incisors (5.8%), and mandibular lateral incisors (1.2%) as well as maxillary canines (1.2%) (Figure 1).

**Figure 2.**
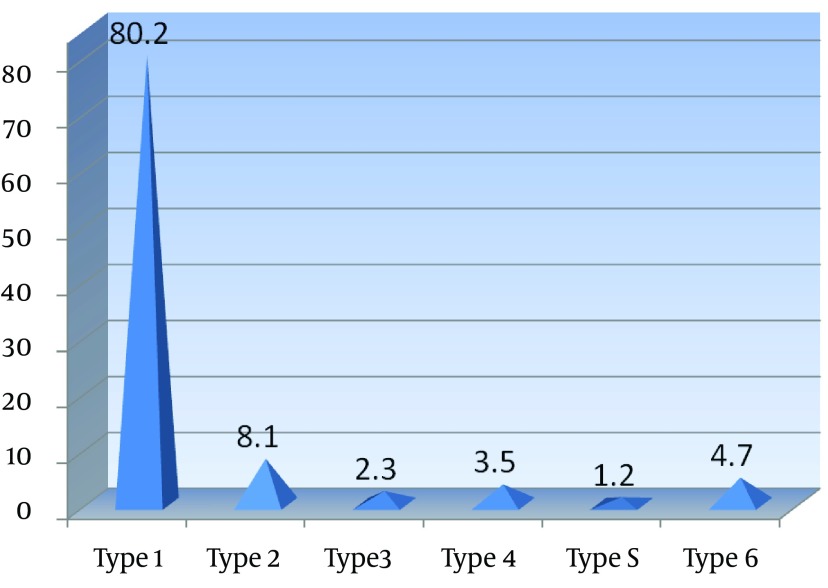
Traumatic Dental Injuries Classified According to Type of Fracture

The most frequent reason for TDI was fall (51.4%) followed by playing sports (41.9%) (Table 1). The majority of accidents occurred at home (58.4%) followed by in school (20.8%) and in the street (18.4%). The most frequent activity of the children at the time of accident were leisure and sports activities. The most common type of injury was Type 1 (enamel fracture) (80.2%), followed by type 2 fracture (i.e. involving enamel/dentin) (8.1%) (Figure 2).

**Figure 3.**
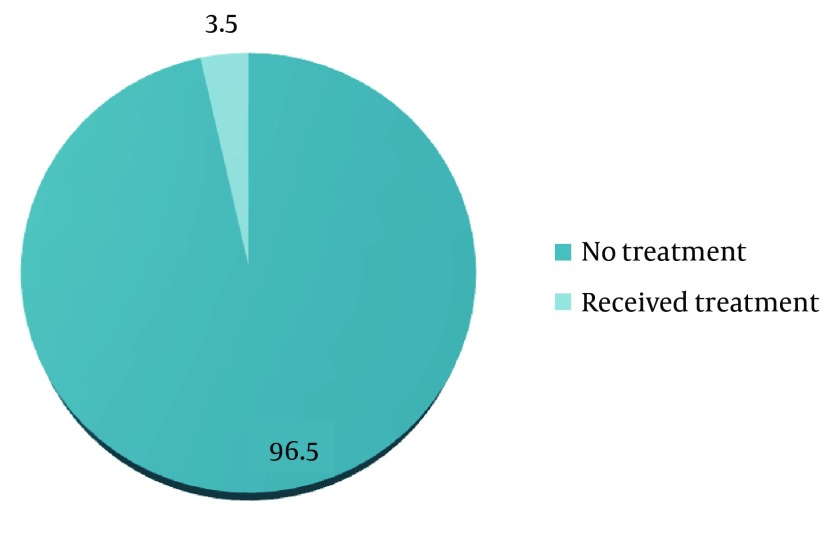
Treatment Received for Traumatic Dental Injuries

Only 3.5% of children with trauma were treated. The type of treatment recorded was acid-etched, adhesively luted restorations, or crowns (Figure 3).

With regard to hypoplastic lesions (Turner’s hypoplasia) on labial surface of permanent incisor teeth, 32% of the children had these lesions while only 3% of children without trauma showed these lesions (P < 0.05). Thus, the children with trauma were at more risk for this type of morbidity. The numbers of injured teeth were more in boys than in girls with the odds ratio of -0.875 (95% CI = -1.358-0.392; P = 0.000) showing negative association between female gender and prevalence of TDI (Table 2).

Children with adequate lip coverage showed lower number of injury (27) as compared to those with inadequate lip coverage (59). In addition, children with inadequate lip coverage were 3.1 times more prone to injuries in comparison to those with adequate lip coverage (OR=3.065, 95% CI= 2.545-3.585, P=0.000) (Table 2). Hence, inadequate lip coverage was identified as the most important and an independent risk predictor for traumatic injuries to permanent anterior teeth. The maximum injuries occurred in children with increased overjet (69%) followed by those having normal overjet (17%) with an odds ratio of 2.441 (95% CI= 1.885-2.997, P=0.000). Thus, increased overjet was identified as an important risk factor for TDI in comparison to the normal overjet.

**Table 1.** Traumatic Dental Injuries Classified According to the Causes of Injuries

Cause of Injury	Values ^a^
**Fall**	44 (51.2)
**Violence/ fight in school**	3 (3.4)
**Domestic violence**	2 (2.3)
**Playing sports**	36 (41.9)
**Others**	1 (1.2)

^a^ Data are presented as No. (%).

**Table 2.** Mantel-Haenszel Common Odds Ratio Estimate and Statistical Significance of Gender, Overjet, and Lip Coverage ^a^

Children Characteristics	Total Number	Number of Injuries	With Injuries	Chi Square Value	Odds Ratio (CI) ^b^
**Gender**					-0.875 (-1.358,-0.392)
Boys	415	355	60	13.22	
Girls	395	369	26		
**Overjet**					2.441 (1.885,2.997)
Normal	552	535	17	103.75	
Increased	258	189	69		
**Lip coverage**					3.065 (2.545,3.585)
Adequate	684	657	27	201.13	
Inadequate	126	67	59		
**Total **	810	724	86		

^a^ P value is zero for all characteristics.

^b^ Abbreviation: CI, confidence interval

## 5. Discussion

This cross-sectional study identified a prevalence of 10.2% of TDI for permanent anterior teeth of 12-15-year-old students of Panchkula District. The reported prevalence of TDIs in the Americans, Asians, and Brazilian adolescents ranged from 4.1% to 58.6% (4-8). Studies conducted among adolescent in India reported the prevalence rates ranging from 8.79% to 33.3% (3, 11, 12). The behavioral and cultural diversity may explain differences in findings between countries as well as within a country. Variation in sampling and diagnostic criteria between different studies may also explain differences in findings.

There was a significant difference in dental trauma between genders; in other words, the prevalence was higher in boys than in girls, which was in accordance with previous studies (4 8). The difference by gender is explained by the fact that boys are more inclined towards vigorous activities. Moreover, the restricted behavior of girls enforced by conservative parents due to cultural and social conditions in India can be added to the possible factors.

Most common fracture was type 1 fracture involving enamel and the most commonly involved tooth was maxillary central incisor. This was in concordance to the reports of Patel and Sujan (3) as well as Nik-Hussein (7).

Most of the TDI in the present study were due to falls occurred at home and school, followed by playing sports in the street. This is of special importance for health policy makers seeking to establish prevention strategies to reduce traumatic orofacial injuries. This includes involving parents more closely to control the home environment. The role of the school environment as a determinant of TDI is well-established; in schools with a supportive social and physical environment, TDI is less likely (4).

Unintentional or accidental injuries to the mouth are common and must be distinguished from child abuse by judging whether the history, including the timing and mechanism of injury, is consistent with the characteristics of the injury and the child’s developmental capabilities (13).

The prevalence of treated traumatized teeth was only 3.5%, which was in concordance to the epidemiological studies done in other parts of the world (8, 14-15). This study showed high rate of unmet treatment needs that can be due to lack of adequate knowledge and proper motivation concerning TDI among children, parents, and teachers. It may be compounded further by limitations imposed due to socioeconomic constraints.

Regarding increased overjet and inadequate lip coverage, the finding in the present study was in agreement with previous reports (3, 4, 7). The results of the present study supported previous findings in which reduced incisor protection through lip incompetence increased the likelihood of trauma (5). Therefore, the treatment of increased overjet is a necessary preventive measure for avoiding TDI.

This study has some limitations; since the study was cross-sectional, causal relationships could not be established and the observed association could be due to other unexplored factors. Moreover, trauma was detected visually and without taking radiographs. The children that did not go to school (i.e. 24.9% of children population of Panchkula) were not studied that will limit generalizability of our results.

However, strength of our study was providing an overview of burden of the TDI among children with 12 -15 years old for the first time and it can be a benchmark for future comparisons by the public health personnel and decision makers.

Public interventions are needed to reduce the risk of TDI among children and adolescents in Panchkula. A safe environment at home and in schools as well as the community such as safer playgrounds can help to minimize the risks. In addition, national and local campaigns and programs should increase social awareness about dental injury. Interceptive orthodontic treatment in this age group would be beneficial to reduce the risk of TDI in children with excessive overjet. In addition, specific local laws to regulate the use of safety equipment are necessary.

The results of this study showed that the prevalence of the TDI to permanent anterior teeth in 12-15-year-old-Panchkula school children was relatively high. The main causes related to the occurrence of TDI were falls and playing sport. TDI was associated with gender, overjet, and lip closure competence. There was a great unmet treatment need.

## Appendices

Sample Size Calculation

Step 1: Base Sample-size Calculation

Formula:

$$n=\frac{t^{2}\mathrm{p (}1-\mathrm{p)}}{m^{2}}$$

Description:

n = Required sample size

t = Confidence level at 95% (standard value of 1.96)

p = Estimated prevalence of variable of interest in the project area (8.8% in this case as obtained from prevalence study)

m = Margin of error at 4% (standard value of 0.04)

Calculation:

$$n=\frac{1.96^{2}\times 8.8(100-8.8)}{4^{2}}$$

n = ~ 193

Step 2: Design Effect

The anthropometric survey is designed as a cluster sample (a representative selection of villages), not a simple random sample. To correct for the difference in design, the sample size is multiplied by the design effect (D).

The design effect is generally assumed to be two for surveys using cluster-sampling methodology.

Calculation

n × D = 193 × 2= 386

Step 3: Contingency

The sample was further increased by 5% to account for contingencies such as non-response or recording error.

Calculation

n + 5% = 386 × 1.05 = 405.3˜405

Further, the sample size was doubled to reduce the standard error.

N = 2 × n= 2 × 405 = 810
